# Comparison of xenograft and allograft bone graft for oral and maxillofacial surgical preparation prior to dental implantation: A systematic review

**DOI:** 10.12688/f1000research.163924.1

**Published:** 2025-07-22

**Authors:** Azza A. Abushama, Nourelhoda Alim, Ahmad Mohammed AlTuraiki, Turki Thaar AlQahtani, Noura Turki Alotaibi, Monerah Mohammed AlQahtani, Nwaf Mohammad AlQahtani

**Affiliations:** 1 Department of Preventive Dental Sciences, Dar Al Uloom University, Riyadh, Riyadh Province, Saudi Arabia; 2 Department of Surgical and Diagnostic Sciences,, Dar Al Uloom University, Riyadh, Riyadh Province, Saudi Arabia; 3 Advanced Education in General Dentistry, Dar Al Uloom University, Riyadh, Riyadh Province, Saudi Arabia; 4 King Khalid University, Abha, Aseer Province, Saudi Arabia

**Keywords:** Keywords: bone graft, xenograft, allograft, sinus augmentation, dental implant.

## Abstract

**Background:**

The implantation of teeth by using dental implants can necessitate the use of bone grafting through xenograft and allograft to increase bone density in the place to induce the integration with the hard tissue. The most commonly used xenografts are usually bovine or porcine and are used in oral and maxillofacial surgery and allografts are those that are taken from the human cadaveric tissue.

**Objectives:**

The aim of the study was to compare clinical usefulness of xenografts and allografts utilized in the front of dental implant placement in adult individuals experiencing oral and maxillofacial surgical procedure.

**Method:**

The systematic search of PubMed and the Cochrane Library (2016 2024) on terms: bone graft AND (xenograft OR allograft) AND dental implant was used. The studies of adults using human xenografts versus allografts in English and published in English were included. Studies that use chemotherapy, cancer, heavy smoking, autografts, and use of animal models were avoided. The quality of studies was estimated with the Cochrane tool by two reviewers who screened and evaluated it.

**Result:**

12 of the 340 studies that included 395 patients (mean age 40–46; 29.9% male, 70.1% female) satisfied the inclusion criteria. Bovine allografts (41.9%) and bovine xenografts (58.1%) were among the grafts utilized. Alveolar ridge preservation (3), delayed (2), immediate (6), and sinus augmentation (2) were among the procedures. The sinus floor, premolars, anterior maxilla, and posterior mandible were frequently used as graft sites. A lack of demographic diversity and small sample sizes were among the limitations. The buccal wall dehiscence and aesthetics in anterior sites require more research.

**Conclusion:**

According to the findings of this systematic review, the advantages of synthetic xenografts like Bio-Oss and synthetic allografts like PerioGlas are exactly the same.

**Registration:** The review protocol was registered with PROSPERO: International prospective register of systematic reviews (CRD42025641250).

## 1. Introduction

Dental implants also have improved restorative dentistry since they are a reliable means of replacing teeth ^1^; however, the outcome of these implants depends on the incorporation of the graft into the surrounding bone ^2^; bone grafting is a routine step in implant dentistry, especially when bone volume is deficient for implantation. Many investigations on human beings and animals together help elucidate the process of healing in the alveolar ridge after tooth extraction. ^3^ Ridge augmentation is known in dental implant ology in relation to the restoration or improvement of the alveolar ridge that disappears in volume and shape due to tooth loss or extraction. This process forms sufficient groundwork for dental implant placement. ^4^

Some authors use “jumping distance” or “jumping gap” to denote the area between an implant placed right after tooth extraction and the surrounding bone. More importantly, this gap is vital to osseointegration and long-term implant stability. Gaps of up to 2 mm can close by themselves, whereas gaps >2 mm require the use of iliac crest bone grafting material. Preserving the jumping gap is another anatomical parameter that should not be eliminated to encourage de novo bone formation on the buccal aspect of the implant site. Therefore, appropriate management of such a gap is critical for achieving implant outcomes. ^5– 8^ Sinus augmentation is another important pre-implant surgery performed to gain additional bone height below the maxillary sinus to create sufficient bone for implantation in the upper jaw. ^9^

Achondo (2004) indicated that this gap can be filled with different types of bone graft materials, such as autografts, allografts, xenografts, alloplastic grafts, platelet concentrates, platelet-rich plasma (PRP), and platelet-rich plasma consisting of growth factors and bioactive proteins thought to enhance tissue repair and regeneration. ^2, 10^

To achieve better outcomes with dental implants, it is essential to have enough bones to hold and provide stability to dental implants. Although the application of advanced diagnostic techniques has been established, the task of accurately installing an implant with equivalent dimensions to the extracted tooth is still complicated. Regarding the intensity of osseointegration and implant stability, it is still possible to conclude that the peri-implant gap plays an important role. ^11^ There are many options available to address this deficiency within orthopaedics, and they can be divided based on natural grafts, which include autografts, allografts, xenografts, and synthetic alloys referred to as alloplasts. Given the nature of xenografts and alloplasts, they are common because of the differences between the two. ^12^ Xenografts are graft tissues derived from non-human species; one common example is Bio-Oss a commercially available bovine bone that has undergone processing to eliminate nearly all organism parts but includes some natural bone mineral. After heat and chemical treatment, the inorganic phase of the bovine bone remains largely HA and retains the interconnected porosity of the substrate structure described above. ^13, 14^ Bio-Oss is the most studied grafting material of xenogeneic origin; it is essentially biocompatible and has a slow rate of resorption, thus allowing new bone formation. ^15^ The relatively porous structure of xenografts contributes to enhanced capillary formation during osteoblastic proliferation and/or migration. ^10^ Porcine bone graft tissue is another porous anorganic material mainly consisting of calcium phosphate used in granulated forms, and a particle size of 0.25-1 mm to 1-2 mm (Gen-Os ^®^) is obtained by extracting the organic portions of porcine bones. ^11^ Allografts are tissues taken from one person to another and are currently used in periodontal plastic surgery because they offer no donor site morbidity as seen with autografts; however, xenografts are not resorbed over time. ^16, 17^ Acellular dermal matrix (ADM) harvested from human cadaver dermis is a major category of allografts used in soft tissue augmentation. The demineralized freeze-dried bone allograft (DFDBA) is largely composed of collagen and an assortment of proteins, including bone growth and differentiation factors, lodged in BMPs. ^18, 19^

This systematic review will therefore explore and critically analyse published literature in order to provide a definitive answer to the question: what is the comparative evidence on xenografts and allografts in oral and maxillofacial surgery prior to dental implant site preparation Through assessment of clinical, radiographically and histological findings in relation to formation and quality of bone, marginal bone level maintenance, soft tissue healing and gingival and probing index, this review aims at presenting facts to the clinicians so as to enable them to choose on appropriate graft material for different clinical conditions.

## 2. Material and Method

### 2.1 Review development and PICO question

This systematic review was conducted based on these guidelines. In the present systematic review, we applied the PRISMA statement ^20^ and registered a systematic review of the International Prospective Register of Systematic Reviews (PROSPERO) database No. CRD42025641250 ^21^ This systematic review did not require ethical approval. The research model applied in this study is the PICO model, which stands for Population, Intervention, Comparison, and Outcome, as expounded below. P (Population): Patients in oral and maxillofacial surgery oral implant preparation, (I): xenograft bone graft, (C): allograft bone graft material, O (Outcome): success rate in preparation of site for implant placement using dental implants may include: (e.g. clinical, radiographic, and histologic analysis). There are some guidelines for assessment (for example after four months of treatment, immediately after surgery, three months later, after six months). Clinical Success Rate: Positivity was defined as the absence of issues and osseointegration of the implant. The assessment is that follow-up time was required to set the success level of the program.

Histological Analysis: The measurement included in the present study was as follows (a quantitative percentage of newly formed bone and residual graft particles).

### 2.2 Eligibility criteria

**2.2.1 Inclusion criteria**

Human investigations: Any adult patient aged 18 years and above who was chosen for oral and maxillofacial surgery for implant site preparation. In particular, allograft materials have been compared to xenografts. Controlled clinical trials (CCTs). Cohort studies. Case-control studies. Comparative studies. Cohort and case control. For the purpose of the present analysis, we compared only the publications that included the data of the same cohort of patients, and only the manuscript that provided a full and sufficient dataset was considered.

The sources searched only analyzed literature published in the last eight years to ensure that the findings relayed the most current practice in the use of the materials and the identification of findings.

**2.2.2 Exclusion criteria**

Receiving chemotherapy or radiation therapy, patients with cancer, or heavy smokers of more than ten cigarettes daily. Research conducted on animals or tissues in a culture medium. Animal models and tissue cultures. Systematic reviews of literature comparing the effectiveness of graft materials that will not merit the comparative mention of autografts.

Here, we followed any trial in which the quality assessment pointed out had a high risk of bias, as determined using the Cochrane Risk of Bias Tool or the ROBINS I risk of bias tool.

### 2.3 Type of Intervention and Comparisons

Studies that compared and contrasted the management of oral and maxillofacial surgery before implantation using xenogeneic, allograft bone grafts in the same patient were included in the study. Because of the lack of research comparing these two forms of blocks directly, studies on ridge and sinus augmentation have also been conducted.

### 2.4 Data collection

The main measure used to evaluate the performance of allografts compared with xenografts in surgeries related to augmentation of oral and maxillofacial sites prior to implant dentistry was the fate of the graft materials and holding of the implants planted in the augmented areas. The following secondary outcomes were also evaluated: The present study was designed to compare intra- and postoperative success rate of both allografts vs xenografts using the following endpoints: bone gain and resorption in the graft materials, changes in marginal bone levels and histological, clinical and histomorphometric findings. Graft survival was defined as the ability to maintain the graft material at the desired location to achieve implant success during re-entry surgery. For dental implants, only those that did not demonstrate mobility, progressive marginal bone loss, or for which implant failure necessitated removal were considered in the survival analysis.

### 2.5 Sources & search strategy

An electronic search was conducted in three electronic databases (google scholar, Medline/(PubMed) and Cochrane Database) up to January 2024 using the English language, human studies, and publications published since 2016 as search filters. combined with free terms using Boolean operators without filters or restrictions, as follows: Disagreements were settled through discussion or confrontation with the second reviewer.

The bibliographic data search was performed using several MeSH terms and free-text words that were connected using Operators: AND or OR. PubMed: ((dental implant OR dental implantation) AND (bone graft [xenograft] AND graft [Mesh] AND bone [Mesh] OR bone graft) [Mesh]) [text word [text word]; Filter/library: randomized controlled trial-comparative clinical studies; Date-March 2008-; language: English; human. Combined with free terms using Boolean operators without filters or restrictions, any disputes were resolved through confrontation and discussion between the two reviewers.

The bibliographic search consisted of a combination of MeSH terms and free-text words, combined with operators (AND or OR). The keywords used were as follows: Google Scholar: Dental implant OR dental implantation [Mesh] AND bone graft [xenograft] AND graft [Mesh] AND bone [Mesh] OR bone graft [text word [text word]; filters: RCT-Compared clinical studies; 8 years’ studies; Human studies; and English studies.

Data were derived from general study characteristics, population characteristics, and graft and implant techniques. Any issues that arose were resolved through discussions with all members involved in contributing to the discrepancy.

### 2.6 Study selection and screening methods

Methods Used in Selecting and Screening of Studies Two reviewers, A. Abushama and N. Alim, conducted a preliminary screening of the articles found using both electronic and manual methods focusing on the titles and abstracts. They then assessed the whole text of the articles that were included or those that did not provide clear information that they should be excluded from the title and abstract.

### 2.7 Clinical data extraction

Within each study, data extraction was performed by the same two reviewers. In situations where some data were missing or not presented at all in the article, the authors of the specific article were asked for additional information. If questions were raised, data that may form that particular point of the question were omitted until more information was collected.

### 2.8 Quality assessment and risk of bias

The quality of the study was independently evaluated by two authors of the present publication, A. Abushama and N. Alim, and the risk of bias for RCTs was performed using a revised Cochrane risk of bias for randomized trials tool (RoB 2). ^22^ The parameters included in the tool (RoB2) were as follows: the process of randomization, the degree to which actual care delivered departs from planned care, the amount and nature of missing outcome data, the measure used to assess the outcome, and the choice of outcomes reported. If all parameters were filled with low risk (green), or up to position two if there was an unclear (yellow), the general result was a Low Risk of Bias (green). For results with risk of bias only in one of the criteria (red) and a maximum of two in doubt (yellow), the result was a Moderate Risk of Bias. However, if the alert value was equal to or included two or more high-risk (red) and/or more than two unclear risks (yellow), then the result was a High Risk of bias.

The ROBINS-I tool was used to evaluate the risk of bias in non-randomized studies of interventions. ^23^ This tool is designed to evaluate seven domains of bias: confounding bias, participant selection bias, intervention classification bias, intervention implementation and receipt bias, attrition bias sample bias, and sources of the reported result in each domain. The study is categorized further into a group of low risk, moderate risk, serious risk, or critical risk of bias. The global estimate of the risk of bias was generally equal to the higher value declared by each domain.

## 3. Result

### 3.1 Study selection

**Figure 1.**
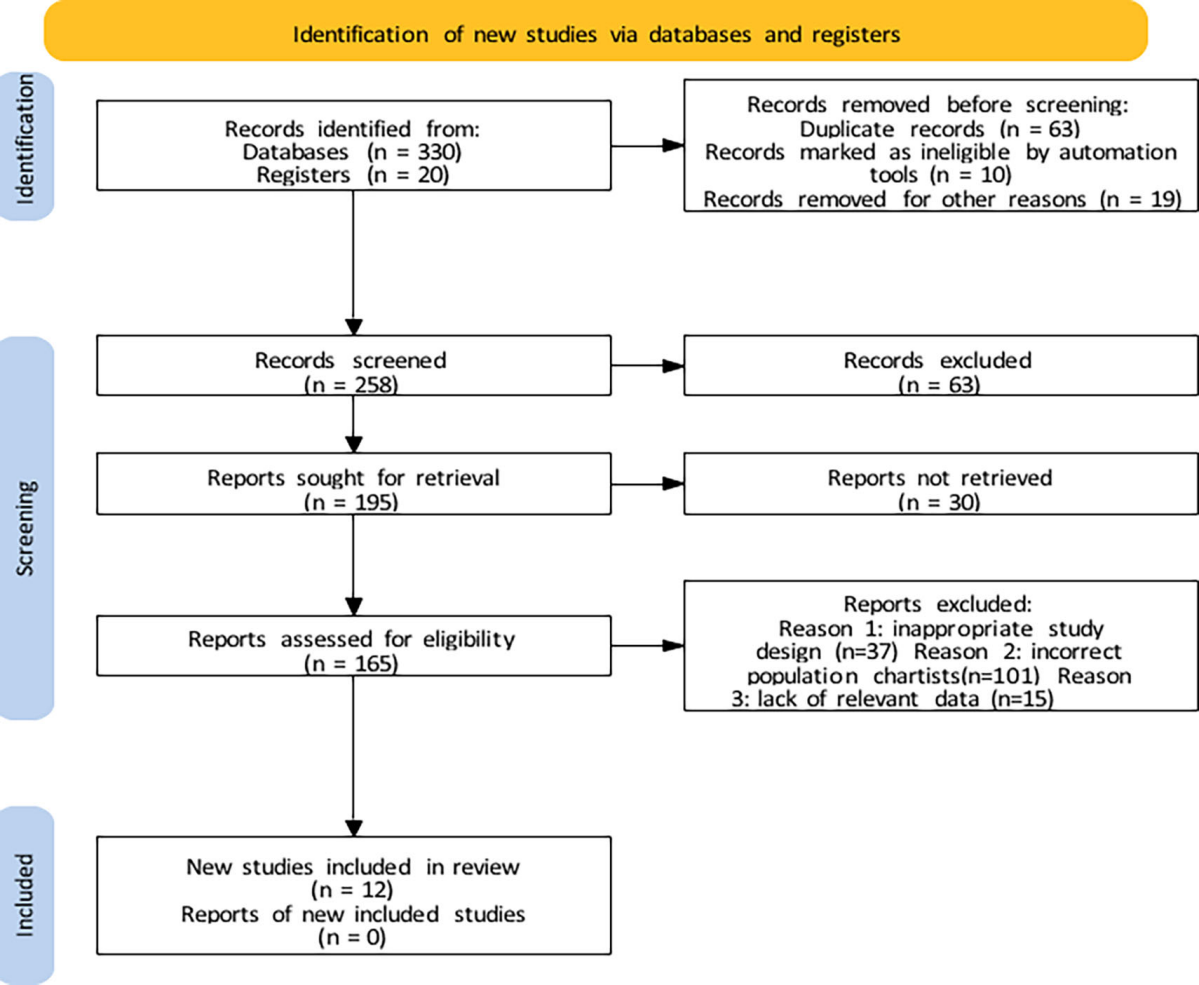
PRISMA flow diagram for study identification and selection.

The electronic search provided 330 papers, of which 20 were found from the manual search 350. Of these, 350 were duplicates and triplicates that were excluded, 258 After the prior screening based on the title/abstract, 165 articles were retrieved for full-text review. Finally, 12 of these studies were included in the review; six involved immediate implant, two delayed implant, three ARP, one sinuses. Figure 1 illustrates the search and selection processes. Meta-analysis was not possible because of the variability in the included studies.

### 3.2 Study characteristic

Of the 12 included trials, Nine (75%) were conducted at universities. ^4, 15, 10, 24– 29^ Two studies (16.67%) were conducted by an independent research clinic and another by a clinical author institute research organization, ^30, 31^ and one study (8.33%) did not indicate the location of the study. Mean age: 40,46 years Thus, the Total number of patients was 395. The gender split of participants was 29.9, male and 70.1, female; nonetheless, 50% of the studies failed to report patient sex. The Total number of bone grafts was 58.1% bovine bone blocks and 41.9% allograft bone blocks.

Specificity of surgery was mentioned by 58.3% of the articles, as the surgeries performed included surgeries involving specified zones of the posterior mandibular region, maxillary anterior region, and the premolar region, sinus floor augmentation, anterior maxillary region, single-rooted teeth region, lateral window approach for the maxilla, posterior molar, and preliminary zones. Nevertheless, 41.7% of the studies investigated failed to specify them for surgical sites. A wide variety of bone grafts were used, with some materials: 8.3% used a 10% collagen matrix, 8.3% used a collagen membrane, 8.3% used NBCM and RECXC, and 75% of which the response did not specify which type of membrane they employed.

### 3.3 Outcome evaluation

Comparing the Gingival Index for FM of the two study subjects at 1 year, we found the following results: allograft 0.86 ± 0.31, and xenograft 0.82 ± 0.12. In the IMP site at 1 year, AL adjusted for Allograft resulted as 0.94 ± 0.21 and xenograft 0.80 ± 0.26 for both groups for the maintenance of gingival health. Probing depth: Consequently, the values in both groups declined over the 1-year period, implying that good implant stability was achieved. Bone resorption: Both groups experienced a mild degree of bone loss around the implant in the mesial and distal areas, although the results were not statistically significant. ^15^ The average contrast and differential entropy of the allograft and xenograft image pairs were carefully compared, and no significant differences were observed (P > 0.05). Furthermore, the authors could not document a significant difference in the dif variance between xenografts and allografts. Therefore, all other of the following pairing; X-Atl, X-PT, X-Pal, Pt-Atl, Pt-Pal and Atl-Pal had a significantly different outcome (P < 0.05). ^24^ Radiographic evaluation: Preoperatively alveolar bone loss in the crystal region was measured to be -1.85 ± 1. 26 mm in the xenograft area and -1.75 ± 1. 51 mm at the allograft area (P = 0.791). The distance of the regenerated osseous crest at 3 months following tooth extraction and ridge preservation was 1.17 ± 0.83 for the group treated with xenograft and 1.00 ± 1.14 for the group treated with allograft (P = 0.523). In the last re-entry session, the sites with grafted bone differentiated them again at 6 and 8 months postoperative. After the radiographic assessment, the mean bone resorption of the allografts appeared to be slightly greater than that of the xenografts 0.9 ± 0.52 mm after 3 months. Change in bone loss after these months for allografts was raised to 1.83 + 0.42 and for xenografts was 1.37 + 1.12 with no significant difference, P > 0.5 ^30^ Clinical comparison The assessment of gingival and plaque indices The gingival and plaque indices in the two groups were compared statistically at three, six, and 12 months of follow-up in the study groups demonstrated no statistical significance (P > 0.05). The mean probing depths and resorption of bone at three, six, and 12 months of follow-up were not statistically significant (P > 0.05) in the intergroup comparison. ^10^ A clinical and histomorphometric analysis of both groups showed shrinkage of bone dimensions. At mesial, canter and distal sites, the vertical changes in dimension were 20.6, 0.5, and 20.1 mm for the allograft and 21.1, 20.4, and 20.9 mm for the xenograft. The horizontal changes in dimensions were 21.4 mm for the allograft and 22.6 mm for the xenograft. New bone and residual graft material were 25.5610.1% and 33.869.4% at the allograft and 35.3616.8% and 22.2613.4% at the xenograft sites. ^4^ Xenograft in the immediate implant site showed excellent osseointegration around the immediate implant site. However, the differences between the groups were not statistically significant. ^25^ A non-significant difference was observed in all parameters at different time intervals (P > 0.05) recorded on the mesial, distal, buccal, and lingual sides. ^32^

Xavier SP Found Implant survival rate: FFB (fresh frozen bone allograft) 97.8%, BBM (bovine bone mineral) 100% (P = 0.352) Bone volume and resorption (CT evaluation): FFB group: median resorption rate 31.2% BBM group: median resorption rate 12.22%, significant differences in initial and final bone volumes (P = 0.015) and resorption rates (P = 0.009); histological findings: FFB: residual FFB with empty osteocyte lacunae, new bone formation, and no inflammation BBM: particles in close contact with new bone, osteoclasts present, and no inflammation. ^26^ The mean marginal bone level in xenograft at baseline (13.58 ± 1.09), 3 months (12.64 ± 0.88), 6 months (12.02 ± 1.42), and 12 months (11.20 ± 1.26), respectively. In allograft, the marginal bone level was at baseline (14.22 ± 0.26), 3 months (13.52 ± 1.28), 6 months (13.10 ± 0.32), and 12 months (12.12 ± 1.26), respectively. A statistically significant difference was observed between the two groups. Moreover, there were no statistically significant differences between the groups.

Duration of intergroup comparison of the mean marginal bone level. The mean difference of implant stability in xenograft the implant stability was 188.6 ± 22.5 and in allograft was 191.5 ± 18.2, and there was no statistically significant difference found between the groups ^27^ Changes in ridge width at 6 months were 1.5 mm for AL versus 2.5 mm for BB and 2.3 mm ^28^ Newly formed bone percentage: Allograft: mean 65% (range 22-86%) Xenograft: mean 45% (range 13-67%) Significant difference found between allograft and xenograft (P < 0.05) Remaining graft particles percentage: Allograft: mean 27% (range 7-62%) Xenograft: mean 41% (range 10-79%) Distribution analysis: Only the allograft group showed differences in the distribution of newly formed bone percentage (Shapiro-Wilk test) ^29^ Primary endpoint of Scheyer ET: at 6 months, extraction socket horizontal measures were significantly greater for DBBMC + NBCM (average 1.76 mm greater, P = 0.0256). Secondary and Exploratory endpoints: lingual and buccal vertical bone changes were not significantly different between the two treatment modalities, histomorphometric% new bone and % new bone + graft were not significantly different, but significantly more graft remnants remained for DBBMC at 1 month, incision line gaps were significantly greater and more incision lines remained open for DFDBA + RECXC; higher inflammation at 1 week tended to correlate with lower ridge preservation results; and deeper socket morphologies with thinner bony walls correlated with better ridge preservation. Thirty-seven of the 40 sites had sufficient ridge dimensions for implant placement at six months; the remainder were DFDBA + RECXC sites. ^31^

### 3.4 Risk of bias

**Figure 2.**
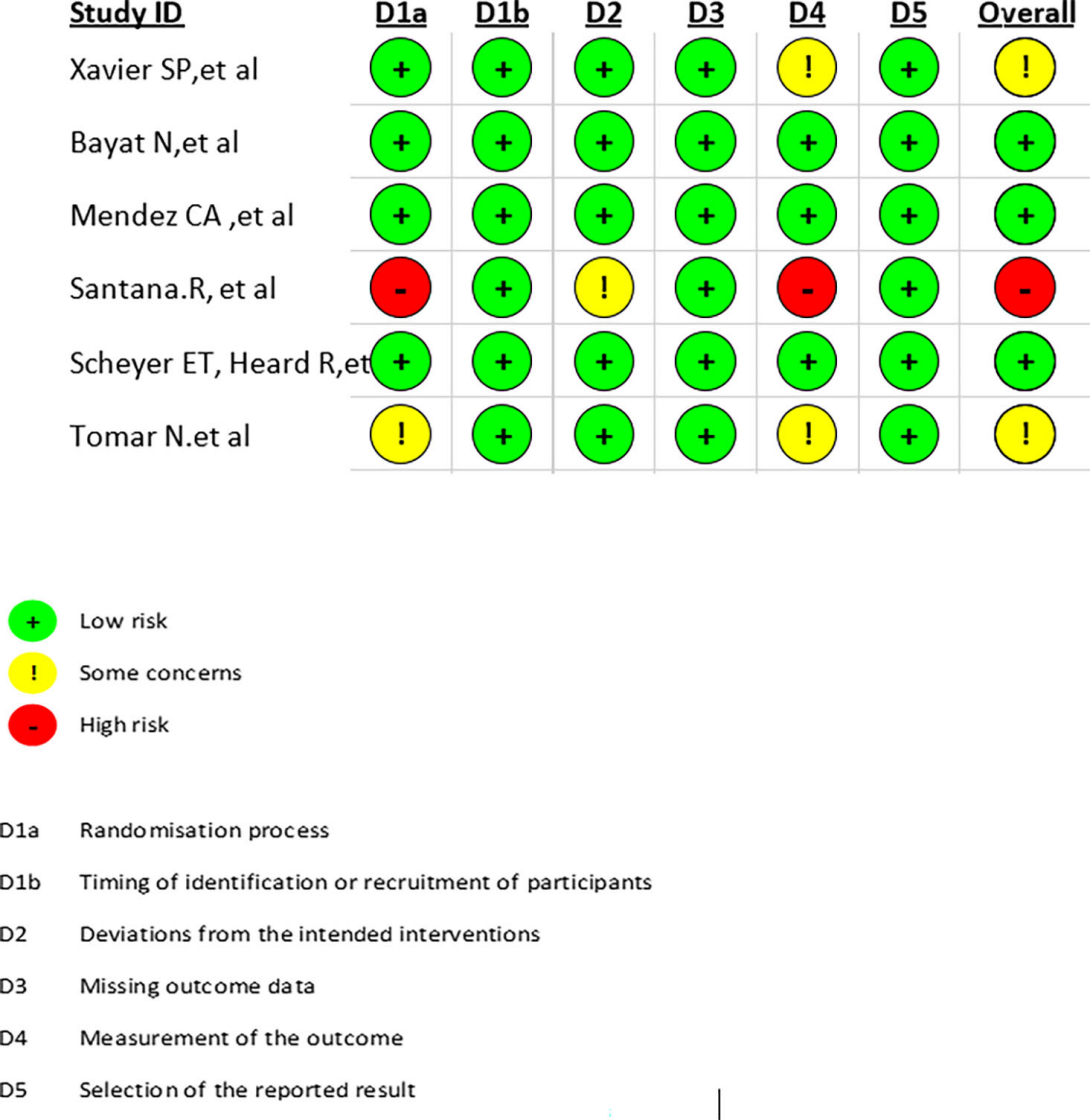
Quality assessment of studies using ROB2 for RCT.

**Figure 3.**
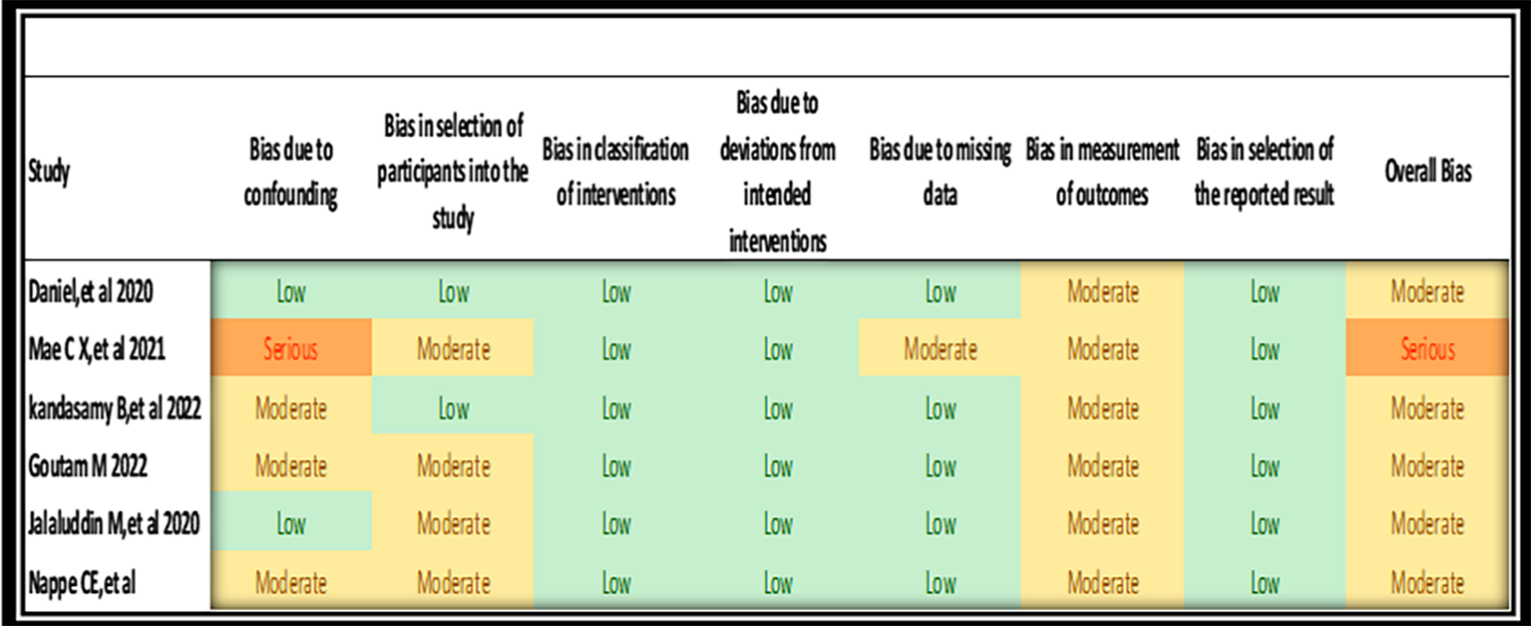
Quality risk of bias using ROBINS I for non-randomized clinical trial.

The majority of domains in the RoB 2 tool ( Figure 2) were low to moderate, indicating that many of the trials making up the review had reasonable rigor. Nevertheless, some concerns and one high-risk domain suggest certain caveats in the interpretation of the findings. In non-randomized studies, there are apparently more serious issues according to ROBINS-I evaluation ( Figure 3). The most worrisome source of risk for bias in this case is confounding, as confounding threatens the validity of findings in observational studies. These results indicate that there are systematic problems in the design and conduct of the studies; more specifically, the moderate risk of bias in participant selection, data selection, and outcome measurement in the five domains is an indication of this.

## 4. Discussion

The studies failed to state patients’ sex which shows favouritism towards female patients The distribution of the graft materials (bovine bone blocks 58.1%, allograft bone blocks 41.9) shows a proper comparison of the xenografts and allografts. Supporting Shibboleths should be included in a comparative assessment. Of (40.46) years, most of the studies were conducted in universities, indicating high research integrity. Nevertheless, the heterogeneity in the study location and the absence of data on the study sites in some contributions underlines the necessity of greater reporting standardization. That only half of the reports did not mention the sex of the patient speaks to its bias towards female patients The choice of graft materials (bovine bone blocks (n = 58.1%), allograft bone blocks n = 41.9% provide a fair comparison between xenografts and allografts. Offer supports the comparative tests … As such, the selection of the sites in this sample suggests that the results should apply across various surgical specialties. However, because site-specific data were not available for 41.7% of the cases, it was impossible to draw site-specific conclusions. The results of the present study for both grafts showed that all of them lost some amount of bone with the help of micro-CT. However, the differences between allograft and xenograft groups were not statistically significant.

This supported the bias in the female patients since the majority of the studies did not include the patient’s sex, and the materials used for grafting (bovine bone blocks//allograft bone blocks) have a balanced comparison of xenografts and allografts. This indicates the need to strengthen the comparative analysis. A study involving different surgical areas implies that many clinical situations may be implicated in the study results. However, 41.7% of studies failed to present specific site information, which caused problems in making site-specific conclusions.

Although both grafts revealed some degree of resorption, the independence of the results obtained in the allograft and xenograft groups suggests that both are moderate in terms of their ability to preserve bone volume. The study findings included increased newly formed bone at allograft sites (65%) as compared to xenograft sites (45%). No such differences were observed in other studies of this type. This variability suggests that it is necessary to determine new bone formation factors in future studies.

Normal cumulative bone implant survival and similar cumulative graft implant stability measurements showed that both grafts provide acceptable substrates for implant placement.

Histological findings provide useful data regarding the biological behavior of these graft materials. Deficiency of fibroosteoid tissue, irregular or vascular connective tissue, and lack of inflammation in both types of grafts were positive findings. BBM sites reveal osteoclast activity, suggesting that bone remodelling is still in progress over the long term. Based on the risk of bias assessment, there are some considerations; for instance, the measurement of outcomes and possible confounding factors. All studies show that both allografts and xenografts are effective treatments for alveolar ridge preservation and sinus grafting; however, it seems that they perform slightly differently depending on the type of application and the assessment metrics used. Both materials exhibit satisfactory osteoconductivity and promote new bone formation and implant integration.

Research investigations to support the effectiveness of both synthetic allografts such as PerioGlas and xenografts such as Bio-Oss in the formation of bone around dental implants are comparable. This was true, irrespective of the augmentation procedure and immediate implant placement. The studies failed to demonstrate any significant differences between allograft and xenograft materials in terms of bone formation, implant stability, marginal bone level, graft integration, and osseointegration, both of which were effective in different clinical situations. ^15, 10, 25, 32, 27^

Even if we have a lesser number of studies and fewer controlled studies comparing, we have studies that estimated long-term consequences proved that both materials had similar results.

This study provides several indications that clinicians may use synthetic allografts and xenografts interchangeably, based on aspects other than satisfactory clinical performance.

That allografts and xenograft materials are ideal for alveolar ridge preservation and sinus grafting. Other researchers have concluded that there is no statistically significant difference in the results obtained using allograft and xenograft materials in the treatment of alveolar ridge preservation. ^4^ In another study, the success rate of bovine bone mineral (BBM) used in sinus augmentation proved to be as successful as that of fresh frozen allograft (FFB).28 The third study consisted of an analysis of allograft material with a PEG barrier and found that this procedure preserved ridge width better than BBM and blood coagulum with only a PEG barrier. ^28^

New allografts were associated with significantly larger volumes of newly formed bone than were xenografts. ^24^ However, a high rate of osseointegration and formation of new bone has been reported with both FFB and BBM in sinus augmentation. ^26^ However, one study reported comparable histological characterization as well as expression of mineralized tissue in the allograft and BBM groups.

Study Limitations and Future Research: Although these studies provide important findings, the authors have recognized the drawbacks in their research, including small group size and larger, less heterogeneous samples in future investigations. Research on anterior sites where aesthetics is more important, considering buccal wall dehiscence defects as a critical assessment of biomaterials and procedures.

## 5. Conclusion

Finally, based on the cumulative findings of these studies, it can be ascertained that synthetic allografts such as PerioGlas and xenografts such as Bio-Oss are equally effective and reliable GBR biomaterials in dental implant surgeries. These similar results enable clinicians to have several choices in the course of treatment, since the graft material source can be selected based on other considerations if the bone grafting approaches yield similar outcomes.

## Data Availability

No data associated with this article. Zenodo: Comparison of xenograft and allograft bone graft for oral and maxillofacial surgical preparation prior to dental implantation: A systematic review, https://doi.org/10.5281/zenodo.15674417 ^33^ This project contains the following underlying data: prisma, appendix abd ROBINS.docx PRISMA_2020_abstract_checklist.docx PRISMA_2020_checklist (1).docx Data are available under the terms of the Creative Commons Attribution 4.0 International license (CC-BY 4.0).
