# Supplementary material for: Comparison of xenograft and allograft bone graft for oral and maxillofacial surgical preparation prior to dental implantation: A systematic review
Source: F1000Res. 2025 Jul 22;14:718. [Version 1] doi: 10.12688/f1000research.163924.1 (PMC12423620; doi:10.12688/f1000research.163924.1)
Supplement: Peer review history

# Reviewer report: Reviewer response for version 1

**DOI:** 10.5256/f1000research.180345.r405813

**Published:** 2025-09-10

**Authors:** Vaibhav Awinashe

**Affiliations:** 1 Qassim University, Buraydah, Saudi Arabia

This Systematic Review has been done with the aim at presenting facts to the clinicians so that they can utilize this review to choose on appropriate graft material for different clinical conditions during dental implant procedure. The study is good and all protocols have been meticulously followed. Kindly find  below my comments

 1. Objective- I think the objective written in the abstract section must be written more clearly. The aim of the study was to compare clinical usefulness of xenografts and allografts utilized in the front of dental implant placement in adult individuals experiencing oral and maxillofacial surgical procedure. This is not clearly understood.

 2. Research Question& Protocol - Research question is clear and protocol is well developed and followed

 3. Literature search - the search is comprehensive and full search strategy is reported.

 4. Study selection and screening- inclusion and exclusion criteria are clear.

 5. Quality Assessment - The quality of the study was independently evaluated by two authors of the present publication, Proper tools have been applied.

 5. Data extraction- data extraction has been done systematically.

 6.Limitations- Authors have identified and discussed potential limitation.

 7. Discussion & Conclusion - Discussion and conclusion is well written and supported by presented evidence.

 Hence after the review the article is ACCEPTED OR APPORVED from my side with minor correction in objectives as mentioned

Are the rationale for, and objectives of, the Systematic Review clearly stated?

Yes

Is the statistical analysis and its interpretation appropriate?

Yes

If this is a Living Systematic Review, is the ‘living’ method appropriate and is the search schedule clearly defined and justified? (‘Living Systematic Review’ or a variation of this term should be included in the title.)

Partly

Are sufficient details of the methods and analysis provided to allow replication by others?

Yes

Are the conclusions drawn adequately supported by the results presented in the review?

Yes

Reviewer Expertise:

Prosthodontics &Implant prosthodontics

I confirm that I have read this submission and believe that I have an appropriate level of expertise to confirm that it is of an acceptable scientific standard.

# Reviewer report: Reviewer response for version 1

**DOI:** 10.5256/f1000research.180345.r408954

**Published:** 2025-09-04

**Authors:** Khadeer Riyaz

**Affiliations:** 1 Oxford Dental College, Bangalore, India

The key words should have been mentioned in alphabetical order. It would be beneficial to provide more details about the word 'HA'. The article has been well prepared and mentioned. A flow diagram has been mentioned and well prepared according to PRISMA guidelines. The conclusion needs more elaboration. Since this study has collected up to January 2024, any further reviews conducted have not been mentioned, which makes it a non-living systematic review, as an LSR needs the current reviews published until the end of 2024.

Are the rationale for, and objectives of, the Systematic Review clearly stated?

Yes

Is the statistical analysis and its interpretation appropriate?

I cannot comment. A qualified statistician is required.

If this is a Living Systematic Review, is the ‘living’ method appropriate and is the search schedule clearly defined and justified? (‘Living Systematic Review’ or a variation of this term should be included in the title.)

No

Are sufficient details of the methods and analysis provided to allow replication by others?

Yes

Are the conclusions drawn adequately supported by the results presented in the review?

Yes

Reviewer Expertise:

Invisible orthodontics ,Photobiomodulations with wavelengths,Accelerated tooth movement ,Lasers

I confirm that I have read this submission and believe that I have an appropriate level of expertise to confirm that it is of an acceptable scientific standard.
